# Flexible, Light-Interacting, B-Shaped Structures for Computations

**DOI:** 10.34133/research.0085

**Published:** 2023-03-30

**Authors:** Nan Yang, Zheng Qian, Huaxian Wei, Yubo Zhang

**Affiliations:** Intelligent Manufacturing Key Laboratory of the Ministry of Education, College of Engineering, Shantou University, Shantou 515063, China.

## Abstract

Integrating mechanical computing functions into robotic materials, microelectromechanical systems, or soft robotics can improve their intelligence in stimulation–response processes. Current mechanical computing systems exhibit limitations, including incomplete functions, unchangeable computing rules, difficulties in realizing random logic, and lack of reusability. To overcome these limitations, we propose a straightforward method of designing mechanical computing systems—based on the logic expressions—for complex computations. We designed soft, B-shaped mechanical metamaterial units, and compressed them to render stress inputs; the outputs are represented by the light-shielding effects caused by the unit deformations. We realized logic gates and corresponding combinations (including half/full binary adder/subtractor and addition/subtraction of 2 numbers with multiple bits) and provided a versatile solution for making a mechanical analog-to-digital converter to generate both ordered and disordered numbers. We performed all of the computations within the elastic regions of the B-shaped units; thus, after one computation, the systems can return to the initial states for reuse. The proposed mechanical computers will potentially enable robotic materials, microelectromechanical systems, or soft robotics to perform complex tasks. Furthermore, one can extend this concept to systems that are based on other mechanisms or materials.

## Introduction

Compared with electronic computation, mechanical computation has some merits for applications under extreme conditions, e.g., high radiation or high temperature [1]. Therefore, mechanical computation based on Babbage’s machine from 1837 [2] has recently regained attention in the context of creating smart mechanical devices [3,4]. Researchers have embedded such mechanical computing systems into robotics [5,6], robotic materials [7,8], and electromechanical devices [9,10] as “brains” in a manner that substantially improves sensing–analyzing–responding capabilities for given tasks.

Broadly, metamaterials that feature interactions between unit cells and external inputs can achieve unconventional mechanical [11–13], thermal [14–16], acoustic [17–19], and optical [20,21] properties. As a subclass, mechanical metamaterials exhibit substantial computation potential due to their multiple force–deformation modes. One can extract binary states of “0,1” by various deformation patterns [22], based on which different logic gates can be realized in a volatile or nonvolatile manner [23,24]. Structurally, the computing systems can be origami-based [25,26], kirigami-based [27], curved beam-based [28,29], or cellular structure-based [8]. In computing mechanisms, the systems can be based on monostability [8] and multistability [25,30].

However, there are limitations to some current systems. First, some logic systems that are realized by mechanical metamaterials are not function-complete [25,31], which limits their applications in realizing versatile basic logic gates and logic combinations. Second, the computing rules of some mechanical logic systems are not easily changed [8], which limits such systems’ flexibility in certain applications. Third, some mechanical computing systems did not realize analog-to-digital converters (ADCs) [6,8,28,29,31]; ADCs are also important for the construction of robotic materials with binary-based information processing capacity because the environmental information to be processed is usually not binary. Fourth, some mechanical logic systems cannot return to their original states, since they stay in a different stable state after certain operations, which renders them nonreusable [25,28,30]. Additionally, every designer might face the following question: how to make a specific structure to realize a targeted computing function, which requires a general method. These issues form the basic questions and capability limitations of current mechanical computing systems.

To overcome these issues, we propose a reassemble mechanical computing system by interactions between soft mechanical metamaterial units and light. One can change the locations of these units to realize various computations, including all basic logic gates and logic combinations (half and full adder, and half and full subtractor). Furthermore, we provided a versatile solution for realizing a mechanical ADC to generate both ordered and disordered numbers. The computing processes are based on the elastic deformations of B-shaped soft units; thus, each system can return to its initial state for reuse. Our design is straightforward and general, since one can directly construct a complex computation system that is based on logic expressions for realizing targeted computing rules. Not limited to the interactions of units and light, one can easily extend our design concept to computing systems with other mechanisms and materials.

## Results

### Structural design

**Fig. 1.**
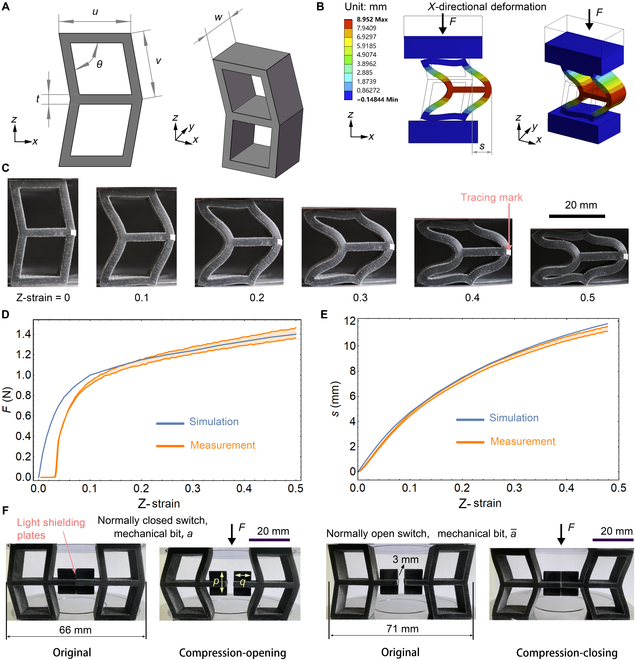
Structural design of the B-shaped structure. (A) Geometry in 2 views. (B) Finite-element simulation of compression at 0.25 z-strain. (C) Experimental photographs at z-strain = 0, 0.1, 0.2, 0.3, 0.4, and 0.5 (see also Video S6). Simulation and measurement results of (D) force vs. z-strain and (E) deformation *s* vs. z-strain, where we obtained the measurement results by 3 duplicate samples. (F) Two B-shaped structures with light-shielding plates acting as a normally closed switch (left: if *F* > 0.8 N, *a* = 1; else, *a* = 0) and a normally open switch (right: if *F* > 0.8 N, $\bar{a}$ = 0; else, $\bar{a}$ = 1; NOT logic).

Here, we created a type of structure from soft materials and thus performed deformations that one can extract as “0,1” states under stress inputs. We designed a B-shaped structure by symmetrically combining 2 parallelograms (Fig. 1A), where *u* = 20 mm, *v* = 18.5 mm, *t* = 2 mm, *w* = 12.5 mm, and *θ* = 80° for easy fabrication and experiment. We 3D-printed the B-shaped structures with a class of soft PolyJet polymers (see Methods). Under compression, the 2 parallelograms generated a shear deformation and yielded an *x*-directional displacement of the rightmost point, *s* (see Fig. 1B; see Methods for simulation). Figure 1C shows the experimental compression of a B-shaped structure with various z-strains; the responding force (*F*) agrees well with the simulation results (Fig. 1D). We used a white tracing mark to obtain the relationship between *s* and the z-strain by image processing (see Methods); we obtained good agreement between the simulations and measurements (Fig. 1E).

In Fig. 1F, we designed a light-shielding plate (rectangle; *p* = 13 mm, *q* = 9 mm) for each pair of B-shaped structures, which makes 2 types of mechanical bits that exhibit different behaviors, *a* and $\bar{a}$, where each mechanical bit consists of 2 B-shaped structures. For *a*, the normally closed switch (NCS), initially the 2 light-shielding plates touch (preventing light passage), but upon compression, the 2 B-shaped structures are apart (facilitating light passage). One can express the basic logic as follows: if *F* > 0.8 N, *a* = 1; else, *a* = 0. In contrast, for $\bar{a}$, the normally open switch (NOS), initially the 2 light-shielding plates are apart (facilitating light passage), but upon compression of the 2 B-shaped structures, they touch (preventing light passage). The basic logic is as follows: if *F* > 0.8 N, $\bar{a}$ = 0; else, $\bar{a}$ = 1 (indicating NOT logic). One can use the basic logic to realize other logic gates and complex computations.

For the NCS, the original distance between the 2 B-shaped structures is around 66 mm, which indicates that the gap between the 2 light shielding plates is zero (Fig. 1F). For the NOS, the corresponding distance and gap are around 71 and 3 mm, respectively (Fig. 1F).

### Basic logic gates

Here, we realize basic logic gates AND, OR, and XOR by using the B-shaped structures, with light-shielding plates interacting with sustaining lasers (parameters of the laser generator: spot diameter, 0.5 mm; wavelength, 650 nm; voltage DC, 2.5 to 5.2 V; and electricity, ≤35 mA), which is the basis of realizing complex computations.

**Fig. 2.**
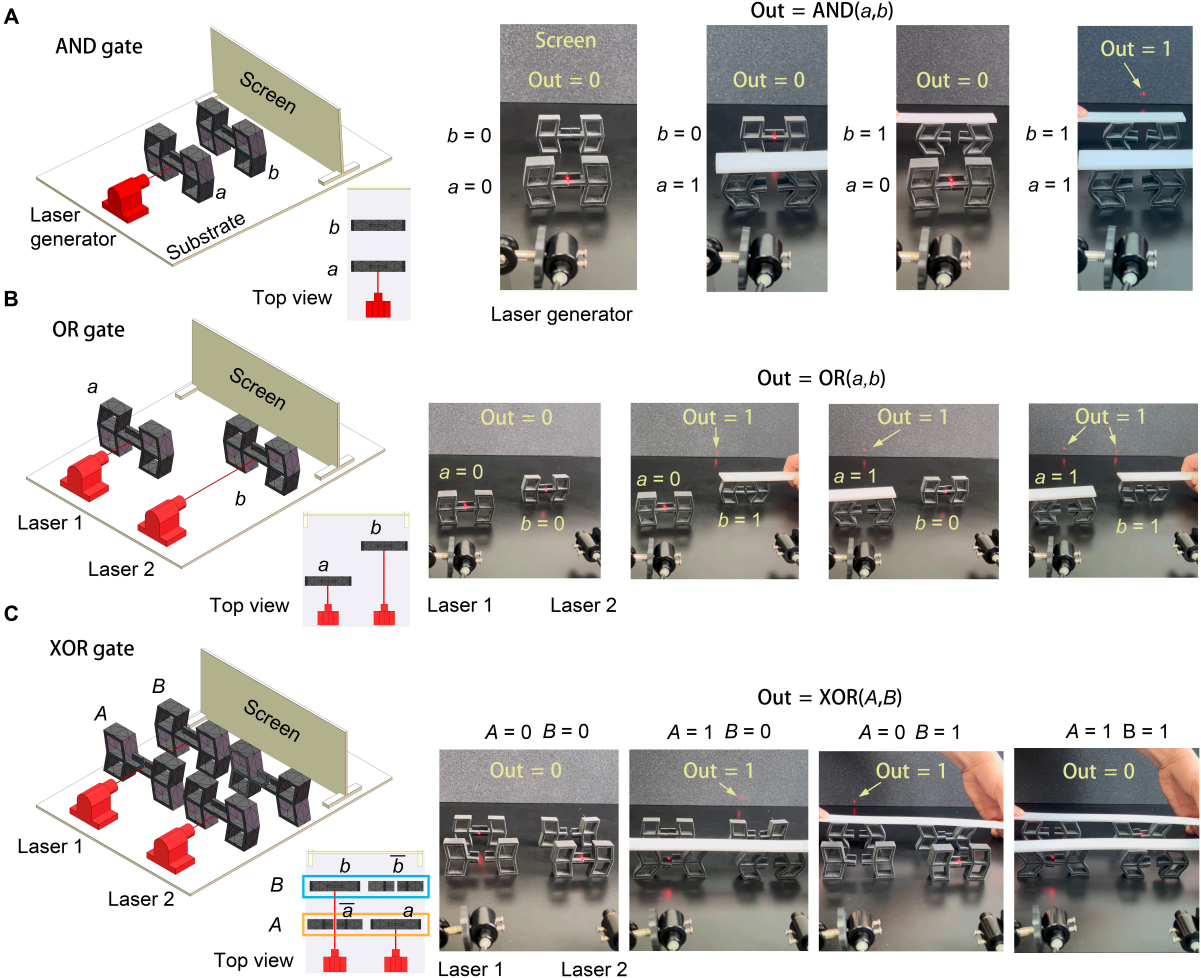
Basic logic gates. (A) AND gate. (B) OR gate. (C) XOR gate. From left to right: system model, top view, and experimental operations. See also Videos S2 to S4.

For the AND gate, we arranged 2 NCSs in series as mechanical bits (*a*, *b*), with a laser generator in front of them (Fig. 2A). If the bit *a* is not compressed, the red laser is forbidden by the light-shielding plate; otherwise, the laser can pass. For example, if one of the bits is not compressed (*a* = *b* = 0, *a* = 1 and *b* = 0, or *a* = 0 and *b* = 1), then the laser beam is blocked; thus, there is no laser spot on the screen behind the bits (i.e., *Out* = 0). Only if the 2 bits are both compressed (*a* = *b* = 1) do we obtain *Out* = 1. Here, we specify that if there is one or more laser spots on the screen (*n* ≥ 1), then *Out* = 1; else, *Out* = 0 for all computations, where *n* is the number of laser spots on the screen. This formulates the logic *Out* = AND(*a*, *b*) = *ab*.

For the OR gate, we arranged 2 NCSs in parallel, with 2 laser generators in front of them (Fig. 2B). Upon compressing one of the bits (*a* = *b* = 1, *a* = 1 and *b* = 0, or *a* = 0 and *b* = 1), the output is *Out* = 1 due to *n* ≥ 1. Otherwise (*a* = *b* = 0), we have *Out* = 0. This realizes the logic *Out* = OR(*a*, *b*) = *a* + *b*.

For the XOR gate, each input consists of 2 switches (see Fig. 2C, bit *A* consists of an NOS $\bar{a}$ and NCS *a*, and bit *B* consists of an NCS *b* and NOS $\bar{b}$). In this paper, the state of *A* is defined as same as the state of the NCS *a*, i.e., *A* = *a*, and *A* = 1 indicates that the switches that correspond to *A* are compressed together (*A* = 0 indicates those switches are not compressed); the same can be said of bit *B*. From the experimental operations, *A* ≠ *B* yields *Out* = 1, whereas *A* = *B* yields *Out* = 0, which realizes the logic $\text{Out}=\mathrm{XOR}\left(A,B\right)=a\bar{b}+\bar{a}b$. See Fig. S2 for realizations of other logic gates: i.e., NAND, NOR, and XNOR. Figure S5 shows some examples of logic combinations.

### Half and full adder

**Fig. 3.**
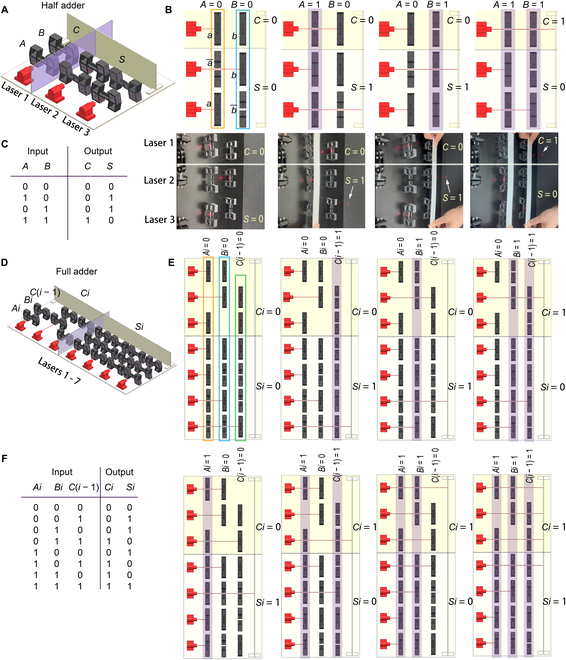
Half and full adder. Half adder: (A) system model, (B) simulations and experimental operations (see Video S5), and (C) truth table. Full adder: (D) system model, (E) simulated operations, and (F) truth table. The purple shades in (B) and (E) indicate that the corresponding input is 1.

A half adder, performing 2-bit addition (*A* plus *B*), has 2 inputs (*A*, *B*) and 2 outputs (*C*, *S*). In the system model (Fig. 3A), we used 3 laser generators, where laser generator 1 corresponds to the output *C*, and laser generators 2 and 3 correspond to *S*. An assumed plane isolates outputs *C* and *S*. In the simulations and experiments, input *A* consists of 2 NCSs (*a*) and 1 NOS ($\bar{a}$), and *A* = 1 indicates that all of the switches that correspond to *A* are collectively compressed (Fig. 3B, the orange frame represents a single input *A*); the same can be said of bit *B* (Fig. 3B, the blue frame represents a single input *B*). *C* = AND(*a*, *b*) denotes the carry bit, and *S* = XOR(*a*, *b*) denotes the addition result. Likewise, *A* = *a* and *B* = *b*. The simulated and experimental results agree well with the truth table (Fig. 3C) for all cases.

Similarly, a full adder (Fig. 3D), performing 3-bit addition [*Ai* plus *Bi* plus *C*(*i* − 1)], has 3 inputs [*Ai*, *Bi*, *C*(*i* − 1)], where *Ai* and *Bi* are 2 addends of the current bit, and *C*(*i* − 1) is the carry from the lower bit. Here, each input consists of 6 switches; input = 1 indicates that the 6 switches are collectively compressed (in each frame, Fig. 3E). There are 2 outputs (*Ci*, *Si*), where *Ci* = OR{AND(*Ai*, *Bi*), AND[*Bi*, *C*(*i* − 1)], AND[*Ai*, *C*(*i* − 1)]} is the carry to the higher bit that requires 3 laser generators, and *Si* = XOR[*Ai*, *Bi*, *C*(*i* − 1)] is the sum result of the current bit that requires 4 laser generators. The simulations for all possible cases (Fig. 3E) agree well with the truth table (Fig. 3F).

### Half and full subtractor

**Fig. 4.**
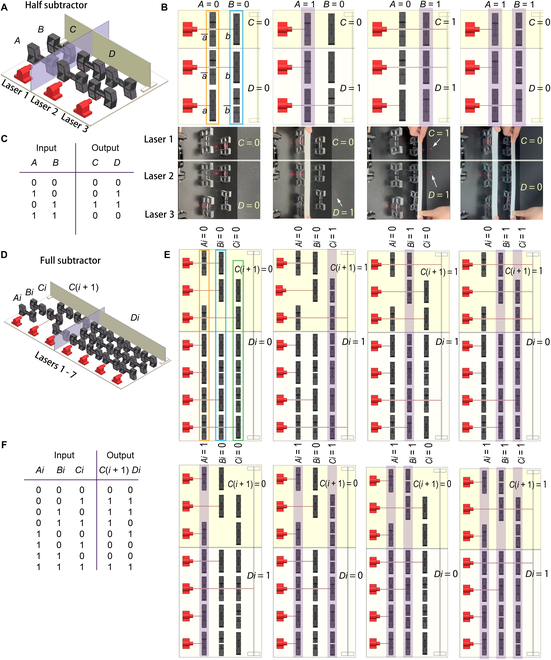
Half and full subtractor. Half subtractor: (A) system model, (B) simulations and experimental operations (see also Video S6), and (C) truth table. Full subtractor: (D) system model, (E) simulated operations, and (F) truth table. The purple shades in (B) and (E) indicate that the corresponding input is 1.

Similarly, a half subtractor, realizing 2-bit subtraction (*A* minus *B*), contains 2 inputs (*A*, *B*) and 2 outputs (*C*, *D*) by using 3 laser generators (Fig. 4A). The input *A* consists of 2 NOSs ($\bar{a}$) and 1 NCS (*a*), and the input *B* consists of 2 NCSs (*b*) and 1 NOS ($\bar{b}$) (see the 2 frames in Fig. 4B). *A* = 1 (or *B* = 1) indicates that all of the switches that belong to *A* (or *B*) are collectively compressed. The output $C=\text{AND}\left(\bar{a},b\right)$ is a comparer (if *a* < *b*, then *C* = 1; else, *C* = 0, where *C* is the borrow bit), and *D* = XOR(*a*, *b*) denotes the subtraction result. Likewise, *A* = *a* and *B* = *b*. The simulations and experimental results (Fig. 4B) agree well with the truth table (Fig. 4C) for all cases.

Further, a full subtractor (Fig. 4D), realizing 3-bit subtraction (*Ai* minus *Bi* minus *Ci*), has 3 inputs (*Ai*, *Bi*, *Ci*), where *Ai* and *Bi* are the minuend and subtrahend of the current bit, respectively, and *Ci* denotes if the lower bit borrows from the current bit. Here, each input consists of 6 switches (in each frame, Fig. 4E).

There are 2 outputs [*C*(*i* + 1), *Di*], where $C\left(i+1\right)=\text{OR}\left[\text{AND}\left(\bar{\mathrm{Ai}},\mathrm{Bi}\right),\text{AND}\left(\mathrm{Bi},\mathrm{Ci}\right),\text{AND}\left(\bar{\mathrm{Ai}},\mathrm{Ci}\right)\right]$ denotes if the current bit borrows from the higher bit, corresponding to 3 laser generators, and *Di* = XOR(*Ai*, *Bi*, *Ci*) is the subtraction result, corresponding to 4 laser generators. The simulations for all possible cases (Fig. 4E) agree well with the truth table (Fig. 4F).

See Section S3 for detailed construction methods using logic expressions for XOR and XNOR gates, the half and full adder, and the half and full subtractor. The full adder/subtractor can be used for the addition/subtraction of 2 binary numbers with multiple bits (Section S4 and S5).

### Design of an ADC

**Fig. 5.**
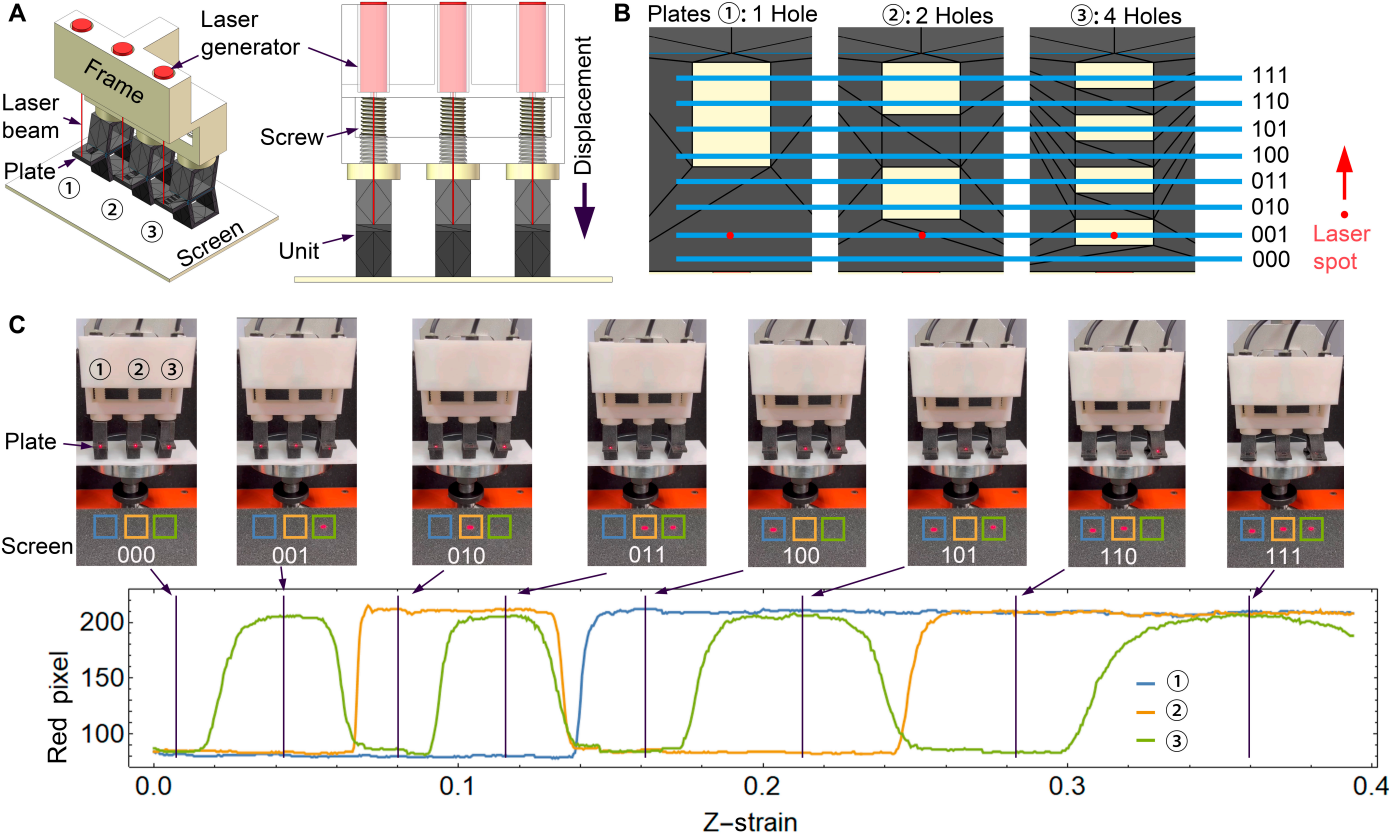
ADC to convert the displacement signals into binary numbers. (A) System diagram (left: 3D view, right: front view with transparent frame). (B) The process is that 3 laser spots scan each hole along each plate (current position: 001 number). (C) The red components of the pixel values of each bit (within each square frame, 32 px × 32 px) vs. z-strain with corresponding experimental photographs, where the colors of the frames and lines are coincident. We obtained the 8 numbers from 000 to 111 in order. Here, we specify that if the red component is larger than 150, then the bit is “1”; else, the bit is “0”. See Video S7.

We designed an ADC to convert the continuous displacement (or force) signals into binary numbers. The resulting binary number was increased with the displacement. In Fig. 5A, a system with 3 identical B-shaped units under a rigid frame is designed to convert the continuously increasing compressive strain into binary numbers from 000 to 111 (i.e., 0 to 7; see Methods for fabrication and Fig. S4 for design), where we set the 3 screws at the same horizontal height. We embedded 3 laser generators into the frame, such that the laser beams can cast onto the horizontal plates of the B-shaped units. In Fig. 5B, the 3 horizontal plates are different: there are 1, 2, and 4 holes for plates ①, ②, and ③, respectively. When one compresses the 3 units, the 3 plates move forward due to the unit deformations, which makes each laser beam scan the holes one by one along each plate. A laser spot that casts on the bottom screen (passes a hole) makes the “1” state; otherwise, the “0” state results. Figure 5B indicates that the 3 laser spots make the number 001. In Fig. 5C, we show the relationship between the red components of the pixel values of each bit and the z-strains with the corresponding experimental photographs of all possible results. In this manner, we sequentially obtained the 8 numbers from 000 to 111 by applying continuous compressive strains from 0 to 0.4.

**Fig. 6.**
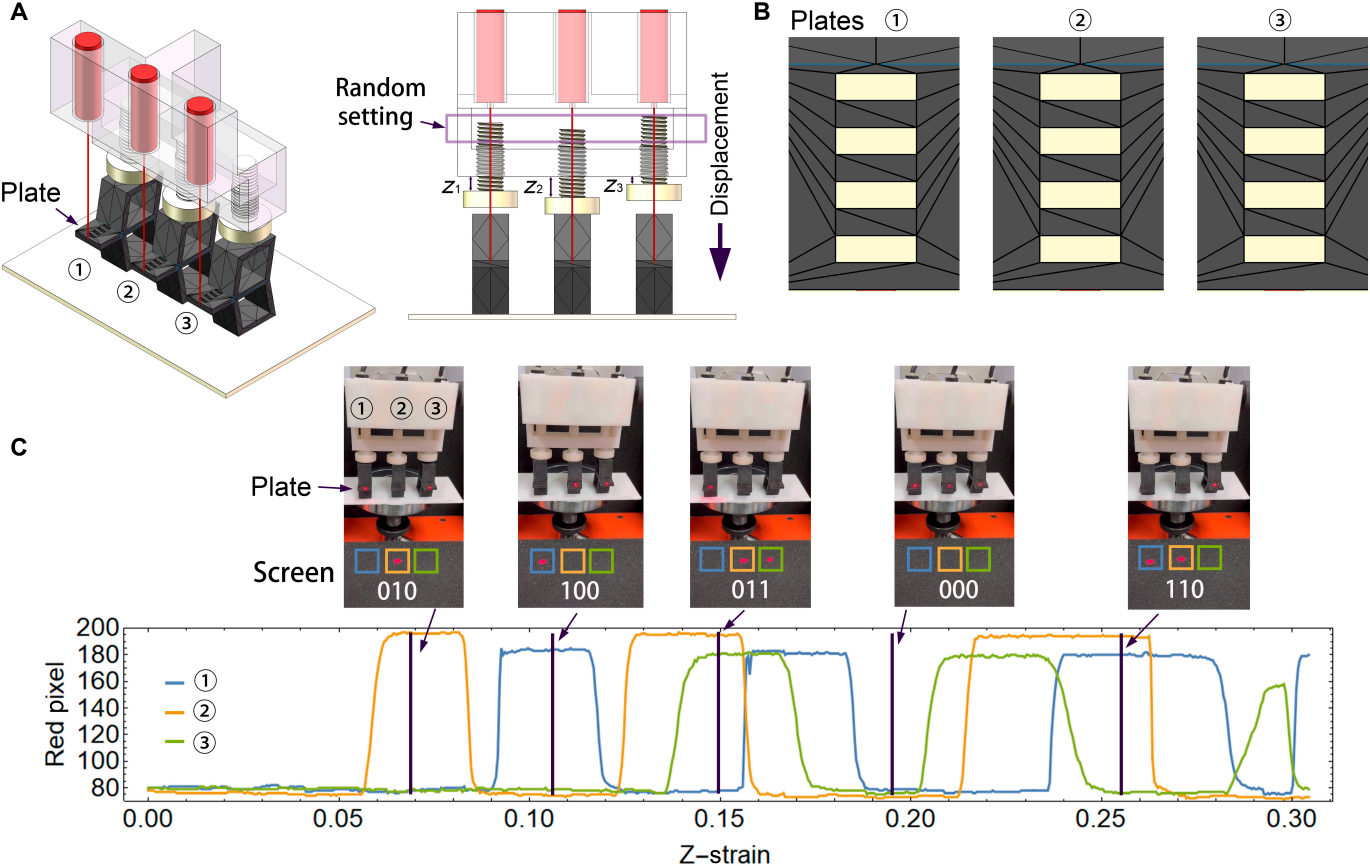
Change of ADC coding scheme. (A) System diagram (left: 3D view, right: front view). Here, *z*_1_, *z*_2_, and *z*_3_ are different. Since the screws compress each unit in different depths, the generated numbers are disordered. (B) The horizontal plates with 4 holes for each unit. (C) The red components of the pixel values of each bit (within each frame) vs. the z-strain with corresponding experimental photographs, where the colors of the frames and lines are coincident. Here, we specify that if the red component is larger than 150, then the bit is “1”; else, the bit is “0”. See Video S8 for all results.

If we set the 3 screws at different horizontal heights (Fig. 6A), the coding scheme between the displacement and resulting number can be changed. Here, we used 4 holes for all plates to make the 1 and 0 states emerge with the same possibility (Fig. 6B), and set *z*_1_ = 5.6 mm, *z*_2_ = 7.1 mm, *z*_3_ = 4.0 mm for the screw positions (Fig. 6A, right). Since the screws compress each unit in different depths, the generated numbers are disordered. Figure 6C shows the red components of the pixel values of each bit changing with the z-strain, where we show 5 disordered results from the 8 possible outputs (000 to 111), such as 010, 100, 011, 000, and 110, which implies a pseudo-random number generator.

## Discussion

There are some merits of the proposed design compared with other works. First, regarding mechanical computing systems based on multistability, after one computing operation, such systems remain in a different stable state and, thus, cannot return to the original state for reuse without an external force [25,28,30]. Our computing systems are based on monostability and, thus, can easily return to the original states with every input being “0”. Second, compared with case-specific methods [8,31], our method to create logic gates is concise and intuitive, and one can build all logic gates by a general approach. In theory, we can realize complex logic combinations, as long as they can be expressed as the disjunctive normal forms of the basic logic gates NOT, AND, and OR. Third, there is no need to redesign the metamaterial structures for logic combinations. In previous work [8], the authors designed 2 2D cellular structures with 5 × 5 blocks (BCs) for OR and NAND logic, but an OR–NAND logic gate combination with 3 mechanical inputs requires a redesigned structure with 7 × 7 BCs, and the conductive channels are completely different from both the OR and NAND logic gates. In our design, one can write OR–NAND logic as $\text{NAND}\left[\text{OR}\left(a,b\right),c\right]=\bar{\left(a+b\right)c}=\bar{a}\bar{b}+\bar{c}$; thus, one can easily build the system model based on the final expression form, by changing the locations and types (convex or concave) of the units (see Fig. S5C; see Fig. S5A, B, and D for other logic combinations), without changing the unit structures. The soft units can be separated from the substrate and reorganized to form other computing rules, since the units were just pasted on the substrate. Here, we used this method to form all the computing devices without fabricating too many soft units.

Moreover, we propose a concise solution for designing a mechanical ADC (Fig. 5) that generates ordered binary numbers under continuous strain/stress, which enables a robotic material to not respond immediately after stimulation but wait for some condition to be satisfied, such as a sufficiently large strain/stress. Accordingly, a different set of the ADC (Fig. 6) can generate disordered numbers, which can be potentially improved as a pseudo-random number generator in the future that enables a robotic material to break away from the current strategy and find a new solution. Besides the heights of the screws, the different geometries of B-shaped units and different sizes of holes (Fig. 6B) can also contribute disordered numbers.

However, there are some limitations in our design that require future improvement. First, the function modularization is not thorough for some logic gate combinations. For example, although one can construct the logic combination OR[AND(*a*, *b*), *c*] = *ab* + *c* by embedding the AND(*a*, *b*) module into the OR gate (Fig. S5A), for AND[OR(*a*, *b*), *c*] = (*a* + *b*)*c*, we need an “optical device” to combine multiple laser beams into one beam and thus ensure modularization (Fig. S5B, left). Alternatively, 2 switches representing bit *C* are required for a nonmodular design {AND[OR(*a*, *b*), *c*] = *ac* + *bc*; Fig. S5B, right}. Nevertheless, for the logic combination $\text{NAND}\left[\text{OR}\left(a,b\right),c\right]=\bar{a}\bar{b}+\bar{c}$, the integrity of the OR (Fig. 2B) and NAND (Fig. S2A) gates is disrupted in the final result by introducing the NOT logic (Fig. S5C). For a mechanical ADC, the laser spot diameter is a key factor that affects the results. If the spot diameter is too large, there will be some unexpected results. For example, the result “111” (Fig. S6, blue spots) might appear between “011” and “100” (Fig. S6, red spots). For improving the pseudo-random number generator, one should incorporate other natural random processes (such as tossing a coin or the uncertain results in electronic latches) into the design concept in the future. Additionally, our mechanical computation systems do not have a memory function, but one can overcome this limitation by combining magnetically mechanical memory systems [22] for realizing complex sequential logics with reprogrammable functions [29]. The optical signals on the output end of the proposed computing system can be converted into other kinds of signals for communication with corresponding devices, such as interaction with electronic devices [32].

## Conclusion

In summary, we formulated a new method for realizing logic gates and corresponding combinations based on the logic expressions, by interactions between soft mechanical metamaterial units and lights. Furthermore, one can integrate such systems to construct half and full adders as well as half and full subtractors for realizing addition/subtraction of 2 numbers with multiple bits. Moreover, we created an ADC to convert continuous displacement (or strain) into ordered binary numbers and generate disordered numbers, respectively. In these systems, the inputs are stresses, and the outputs are optical signals that one can convert into other types of signals—such as electronic—for given applications. Our mechanical computing systems would provide more-complicated stimulation–response functions for robotic materials, microelectromechanical systems, and soft robotics upon fabrication at various scales.

## Methods

### Fabrication of soft B-shaped structures and rigid frames

The soft B-shaped structures and rigid frames were designed with Solidworks software and as STL files that were exported to individual 3D printing systems.

The soft B-shaped structures were fabricated with a Connex object 350 PolyJet System (Stratasys, US) with TangoBlackPlus (soft PolyJet polymers, density: 1.15 g/cm^3^) and a soluble support material (SUP706). The printer had a precision of 16 to 30 μm in the layer direction and 20 to 50 μm in the other 2 directions. In the printer, a UV lamp with an intensity of 1,780 mJ/cm^2^ and a wavelength of 405 nm was used to solidify the polymers. The products were immersed in water for 24 h to remove the support material.

To fabricate the rigid frames, a laser powder bed fusion 3D printing system with nylon powder was used (FS3300PA, powder density: 0.48 g/cm^3^, particle diameter: 10 to 50 μm, fabricated solid density: 0.97 g/cm^3^). A 60-W laser with a spot diameter of 70 μm was applied. The powder deposition thickness, laser scan speed, and hatch spacing were 0.1 mm, 7.6 m/s, and 0.25 mm, respectively. The temperature in the sintering zone was controlled at approximately 167.5°C to minimize the effect of thermal stresses that accumulate in the fabricated part. Sandblasting was used to remove unsintered powder from the products.

### Quasi-static mechanical tests

A 10-kN universal force testing machine (Instron 5984) was used to stretch the TangoBlackPlus base material bars (ASTM standard type 1, see Fig. S6) and compress the B-shaped structures at a constant speed of 15 mm/min. The elastic modulus of the base material, *E =* 0.62 MPa, was obtained in the elastic region with *E* = *FL/*(*ArU*), where *F* is the reaction force of the sample, *L* is the length of the sample, *Ar* is the cross-sectional area of the sample, and *U* is the displacement of the test head along the loading direction.

### Image processing

To measure the *x*-directional deformation *s* (Fig. 1B), a Canon 60D camera was used to obtain videos of the B-shaped structures under compression. The rightmost edge of the white tracing mark of a B-shaped structure in the black background was easily detected. Then, *s* was calculated with a length-to-pixel ratio of 0.049 mm/px.

### Finite-element simulations

Simulation results were obtained with the commercial software ANSYS Workbench19.2. A Mooney–Rivlin material model with 3 parameters was utilized to represent the hyperelastic behavior of the base material, based on ASTM tensile tests. The material constants of *C*_10_ = 89,951 Pa, *C*_01_ = 81,426 Pa, and *C*_11_ = 29,656 Pa were obtained by fitting the test data. Rigid blocks with structural steel material were utilized to simulate the compressing head and substrate (Fig. 1B). The soft modules were meshed by using tetrahedral elements with all of the faces mapped. The rigid blocks were meshed by using uniform hexahedron elements.

To simulate the compression process, 2 rigid blocks were used to contact the top and bottom surfaces of the soft module, with a friction coefficient of 0.7 (see Fig. S9 for the reason). During simulation, the bottom block was fixed in 6 degrees of freedom, whereas the top block was loaded with a translational displacement up to 25% of the module height along the compression direction. The effects of large deformation were considered for the soft modules.

## Data Availability

The data that support the findings of this study are available from the corresponding author on reasonable request.

## Supplementary Materials

Supplementary 1 1. Mechanical test of the base material 2. Construction of NAND, NOR, and XNOR gates 3. Detailed construction methods for XOR and XNOR gates 4. Detailed construction methods for half and full adder, and multi-bit number addition 5. Detailed construction methods for half and full subtractor, and multi-bit number subtraction 6. Force vs. *z*-strain relationships with different friction coefficients Fig. S1. Force test of base material of the B-shaped structures. Fig. S2. Other logic gates. Fig. S3. Detailed construction method. Fig. S4. Design and dimensions of the rigid frame and screws used in the mechanical ADC. Fig. S5. Logic combinations. Fig. S6. Laser spot diameter affects the results. Fig. S7. Operations of *A*2|*A*1 ⊕ B2|*B*1 = *C*2|*S*2|*S*1. Fig. S8. Operations of *A*2 ∣ *A*1 ㊀ *B*2 ∣ *B*1 = *C*3|*D*2|*D*1. Fig. S9. Force vs. *z*-strain with different friction coefficients (simulation).

Supplementary 2 Videos S1. Soft B-shaped structure compressed to 0.5 strain (compression speed: 15 mm/min). Video S2. AND gate operation. Video S3. OR gate operation. Video S4. XOR gate operation. Video S5. Half adder operation (A plus B). Video S6. Half subtractor operation (A minus B). Video S7. ADC to convert the displacement signal into binary numbers. Video S8. Change of ADC coding scheme to generate disordered numbers.
