# Supplementary material for: Flexible, Light-Interacting, B-Shaped Structures for Computations
Source: Research (Wash D C). 2023 Mar 30;6:0085. doi: 10.34133/research.0085 (PMC10065789; doi:10.34133/research.0085)
Supplement: Supplementary 1 — 1. Mechanical test of the base material 2. Construction of NAND, NOR, and XNOR gates 3. Detailed construction methods for XOR and XNOR gates 4. Detailed construction methods for half and full adder, and multi-bit number addition 5. Detailed construction methods for half and full subtractor, and multi-bit number subtraction 6. Force vs. z-strain relationships with different friction coefficients Fig. S1. Force test of base material of the B-shaped structures. Fig. S2. Other logic gates. Fig. S3. Detailed construction method. Fig. S4. Design and dimensions of the rigid frame and screws used in the mechanical ADC. Fig. S5. Logic combinations. Fig. S6. Laser spot diameter affects the results. Fig. S7. Operations of A2|A1 ⊕ B2|B1 = C2|S2|S1. Fig. S8. Operations of A2 ∣ A1 ㊀ B2 ∣ B1 = C3|D2|D1. Fig. S9. Force vs. z-strain with different friction coefficients (simulation). [file research.0085.f1.docx]

**Supplementary Materials**

1. **Mechanical test of the base material**


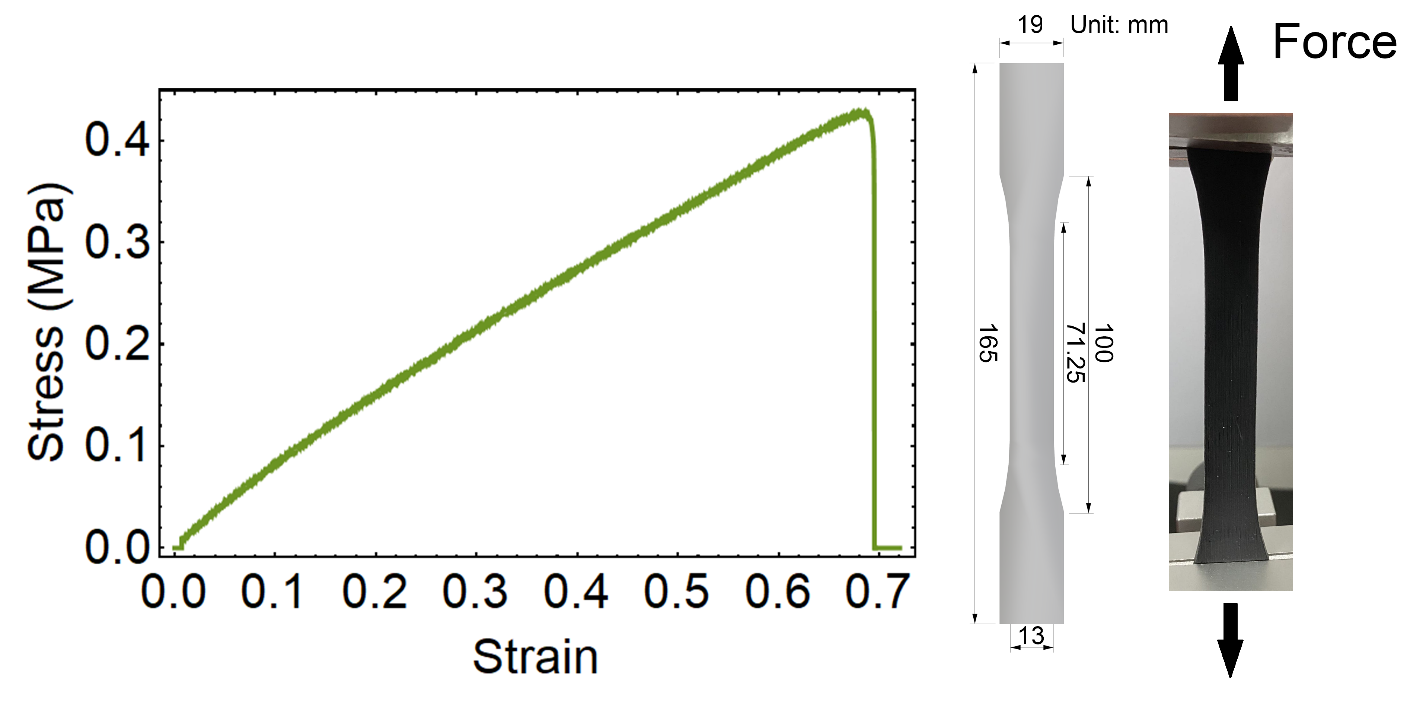


**Fig. S1. Force test of base material of the B-shaped structures.** Stress–strain curve of the base material (TangoBlackPlus), obtained by using a testing ASTM standard type 1 tensile bar with a thickness of 3.2 mm. The testing speed was 15 mm/min.

1. **Construction of NAND, NOR, and XNOR gates**


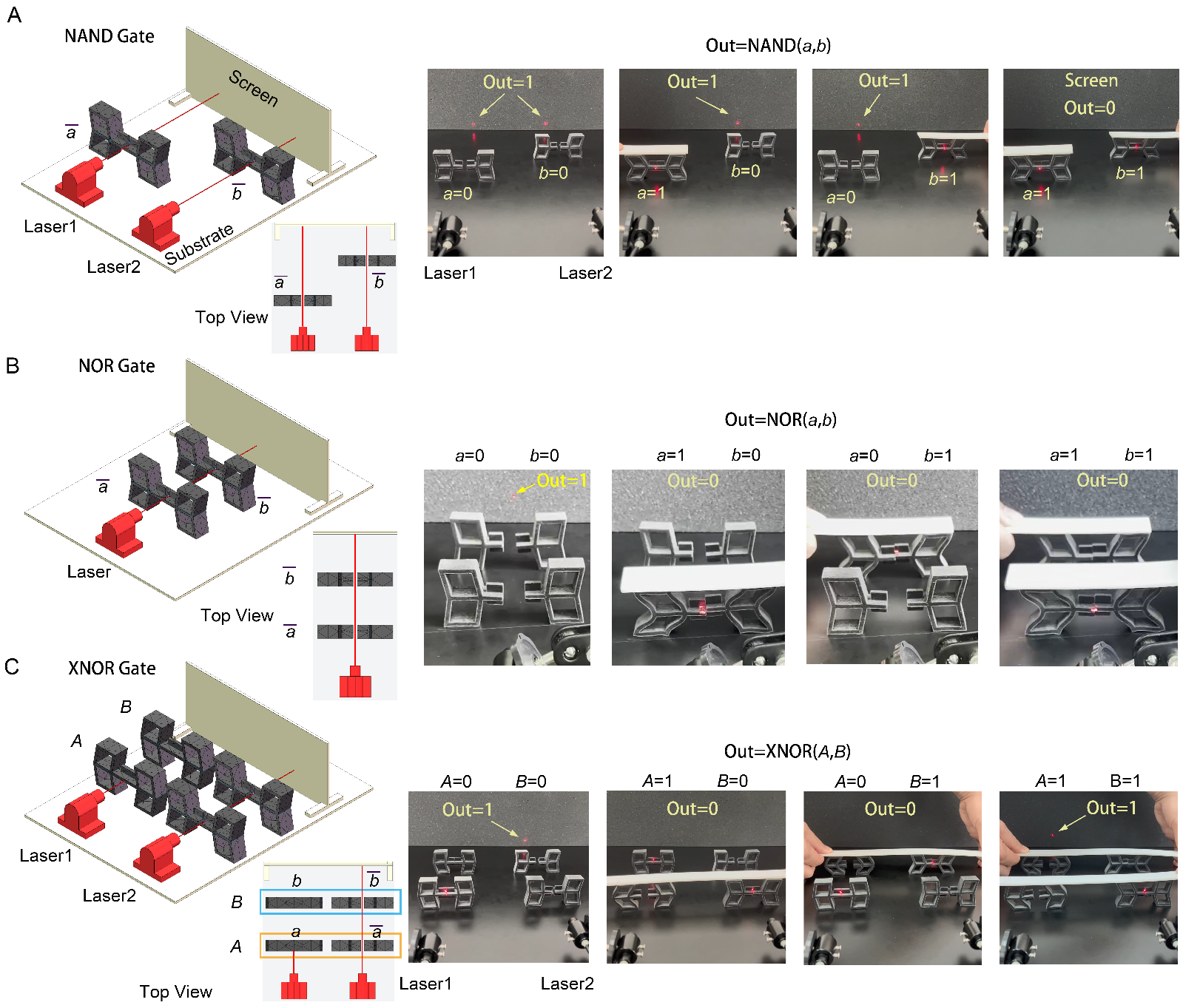


**Fig. S2. Other logic gates.** (A) NAND gate. (B) NOR gate. (C) XNOR gate. From left to right: system model, top view, and experimental operations.

1. **Detailed construction methods for XOR and XNOR gates**

For the XOR gate, an NOS ($\bar{a}$) and an NCS ($a$) are basic components in group $A$, and an NCS ($b$) as well as an NOS ($\bar{b}$) are basic components in group $B$. One can regard groups $A$ and $B$ as input bits; $A=1$ indicates that the switches (basic components or units) that belong to $A$ are all compressed [i.e., the NOS forbids laser passage ($\bar{a}=0$) and the NCS enables laser passage ($a=1$)]. The same can be said of bit $B$. Thus, $A\neq B$ yields $Out=1$ whereas $A=B$ yields $Out=0$; which realizes the logic $Out=\left. \bar{a}b \right.+\left. a\bar{b} \right.=\mathrm{XOR}\left( a,b \right)=XOR\left( A,B \right)$. In this paper, we specify that the states of $A$ and $B$ are as same as those of the corresponding NCSs $a$ and $b$; i.e., $a=A,b=B$. Sign ‘+’ denotes OR operation.

Similarly, for XNOR gate, we have $Out=\left. ab \right.+\left. \bar{a}\bar{b} \right.=\mathrm{XNOR}\left( a,b \right)=XNOR\left( A,B \right)$.


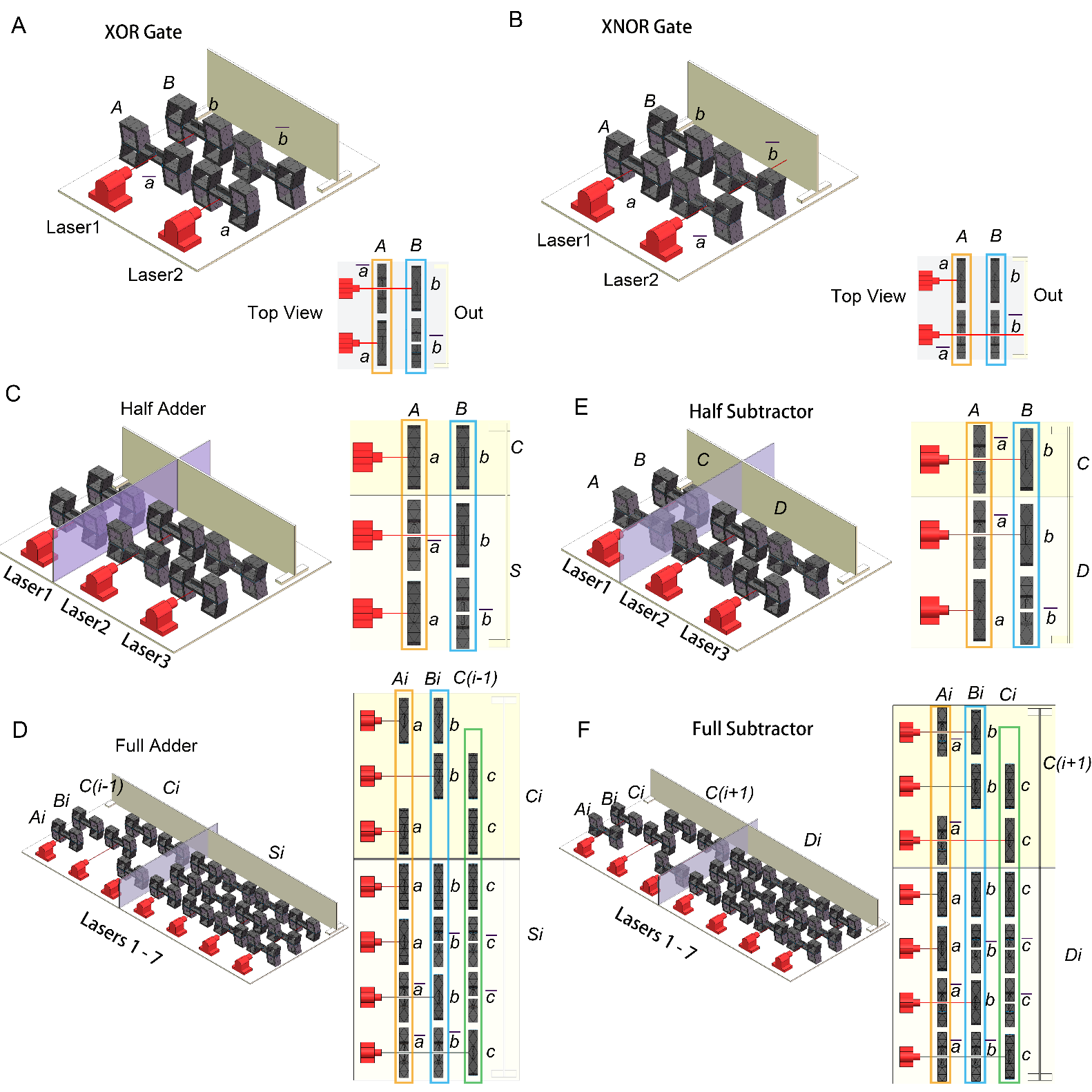


**Fig. S3. Detailed construction method.** (A) XOR. (B) XNOR gates. (C) Half and (D) full adder, and (E) half and (F) full subtractor.

1. **Detailed construction methods for half and full adder, and multi-bit number addition**

For half adder (main text Fig. 3A–C), we have

$$C=ab=\mathrm{AND}\left( a,b \right)=\mathrm{AND}\left( A,B \right)$$

$$S=a\bar{b}+\left. \bar{a}b \right.=\mathrm{XOR}\left( a,b \right)=XOR\left( A,B \right)$$

For full adder (main text Fig. 3D–F), we have

$$Ci=OR[AND\left( a,b \right),\mathrm{AND}\left( b,c \right),\mathrm{AND}\left( a,c \right)]=OR\{AND\left( Ai,Bi \right),\mathrm{AND}\left[ Bi,C\left( i-1 \right) \right],\mathrm{AND}\left[ Ai,C\left( i-1 \right) \right]\}$$

$$Si=\left. abc \right.+\left. a\bar{b}\bar{c} \right.+\left. \bar{a}b\bar{c} \right.+\left. \bar{a}\bar{b}c \right.=\mathrm{XOR}\left( a,b,c \right)=XOR[Ai,Bi,C\left( i-1 \right)]$$

This realizes multi-bit number addition in a recursive manner with $C0=0$. For example, when $i=2$, we have

$$C1=A1B1$$

$$S1=XOR[A1,B1,C0]$$

$$C2=A2B2+B2C1+A2C1$$

$$S2=XOR[A2,B2,C1]$$

which form the result of two-bit number addition by

$$A2\left| A1\oplus B2 \right|B1=C2\left| S2 \right|S1$$

where $\oplus$ and $|$ denote addition sign and isolation sign, respectively. See Fig. S7 for operations.

Generally, when $i=n$, multi-bit number addition can be expressed as

$$A\left( n \right)\left| A\left( n-1 \right)|\ldots|A1\oplus B\left( n \right) \right|B\left( n-1 \right)|\ldots\left| B1=C\left( n \right)\left| S\left( n \right) \right|S\left( n-1 \right) \right|\ldots|S1$$

1. **Detailed construction methods for half and full subtractor, and multi-bit number subtraction**

For half subtractor, we have

$$C=\bar{a}b=\mathrm{AND}(\bar{a},b)=\mathrm{AND}(\bar{A},B)$$

$$D=\left. \bar{a}b \right.+\left. a\bar{b} \right.=\mathrm{XOR}\left( a,b \right)=\mathrm{XOR}\left( A,B \right)$$

For full subtractor, we have

$$C\left( i+1 \right)=\left. \bar{a}b \right.+bc+\bar{a}c=OR[AND\left( \bar{A\dot{i}},Bi \right),\mathrm{AND}\left( Bi,Ci \right),\mathrm{AND}\left( \bar{A\dot{i}},Ci \right)]$$

$$Di=abc+a\bar{b}\bar{c}+\bar{a}b\bar{c}+\bar{a}\bar{b}c=\mathrm{XOR}\left( a,b,c \right)=\mathrm{XOR}\left( Ai,Bi,Ci \right)$$

This realizes multi-bit number subtraction in a recursive manner with $C1=0$. For example, when $i=3$, we have

$$C2=\left. \bar{A1}B1 \right.$$

$$D1=XOR\left( A1,B1 \right)$$

$$C3=\left. \bar{A2}B2+B2C2+\bar{A2}C2 \right.$$

$$D2=XOR\left( A2,B2,C2 \right)$$

which form the result of two-bit number subtraction by

$$A2\left. |A1 ㊀ B2| \right.B1=C3\left| D2 \right|D1$$

where $㊀$ denotes subtraction sign. See Fig. S8 for operations.

Generally, when $i=n+1$, multi-bit number subtraction can be expressed as

$$A\left( n \right)|\left. A\left( n-1 \right)|\ldots|A1 ㊀ B\left( n \right) \right.|B\left( n-1 \right)|\ldots\left| B1=C\left( n+1 \right)\left| D\left( n \right) \right|D\left( n-1 \right) \right|\ldots|D1$$

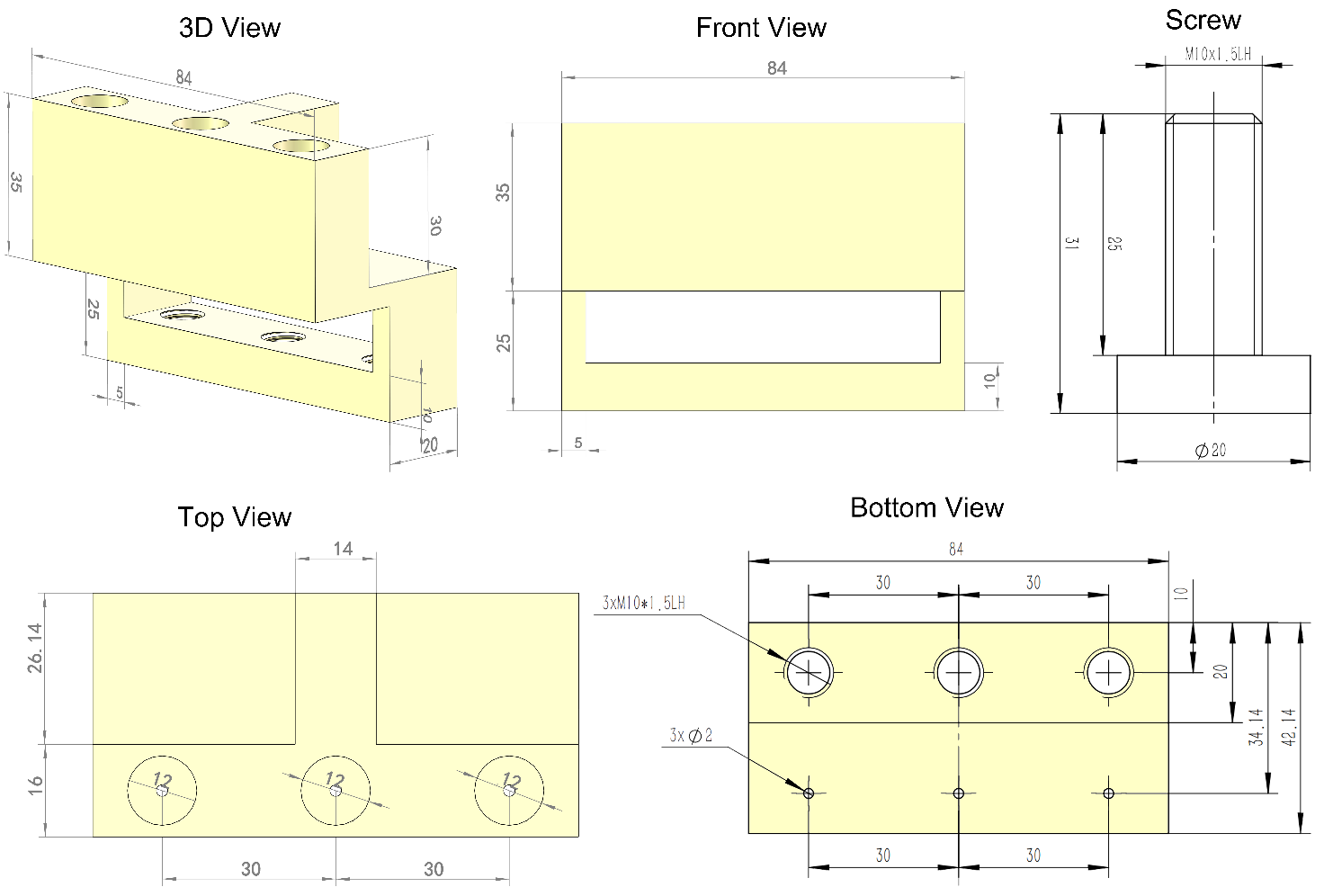


**Fig. S4. Design and dimensions of the rigid frame and screws used in the mechanical ADC.**


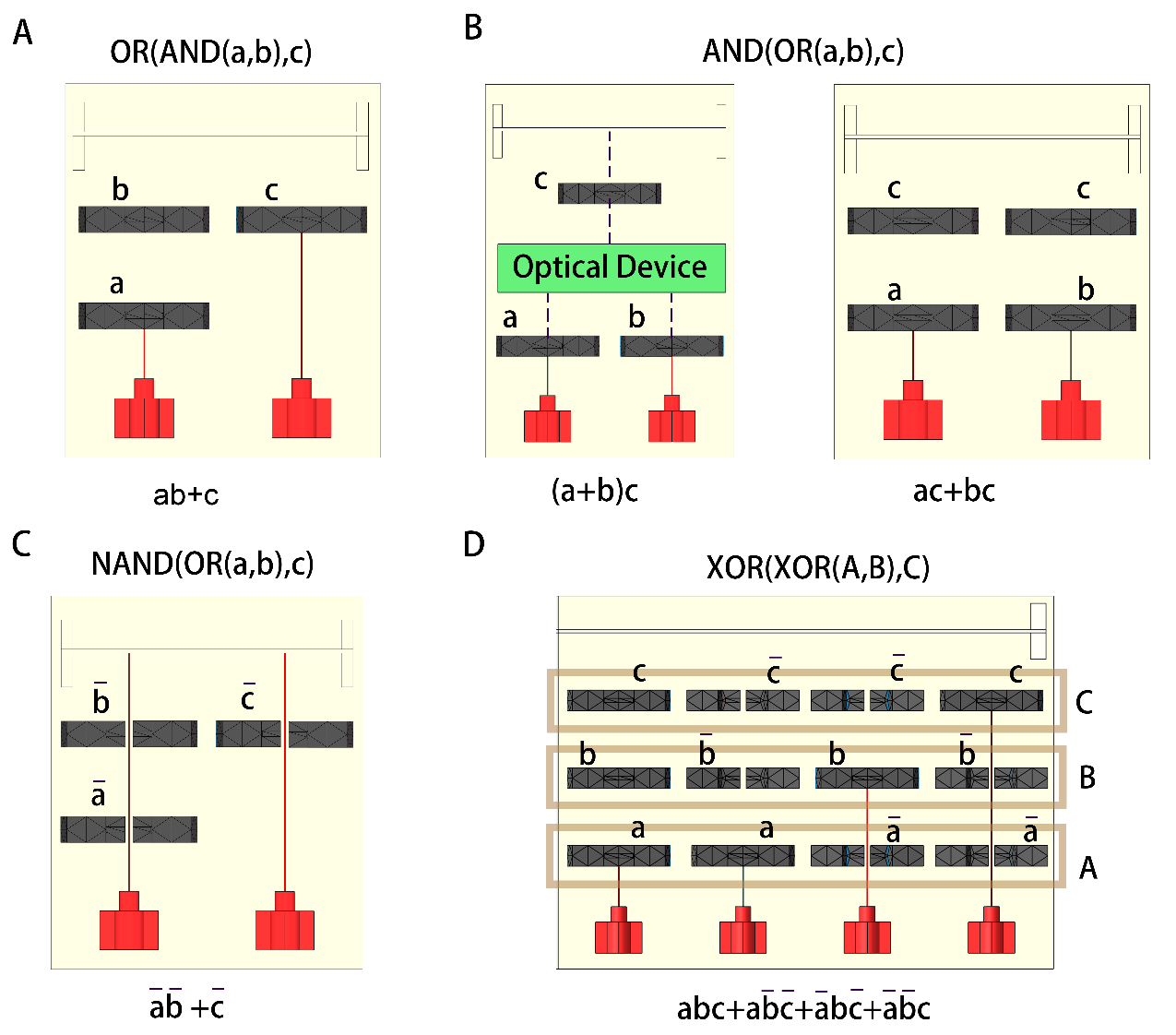


**Fig. S5. Logic combinations.** (A) OR[AND(a, b), c]. (B) Two methods of realizing AND[OR(a, b), c)]. (C) NAND[OR(a, b), c]. (D) XOR[XOR(A, B), C]=XOR(A, B, C).


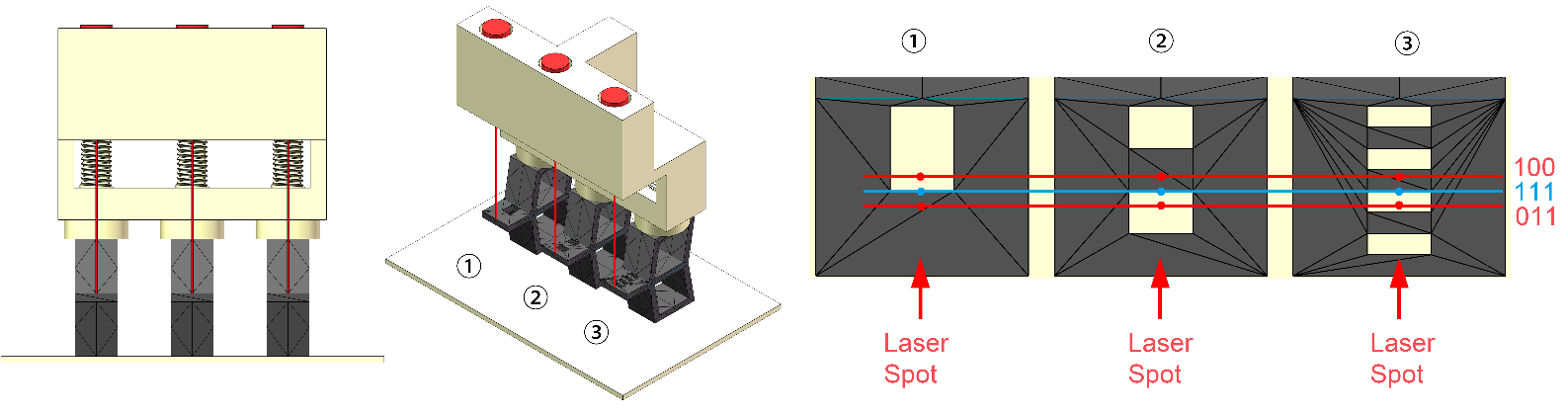


**Fig. S6. Laser spot diameter affects the results.** Here, the result ‘111’ (blue spots) may be evident between the ‘011’ and ‘100’ (red spots).


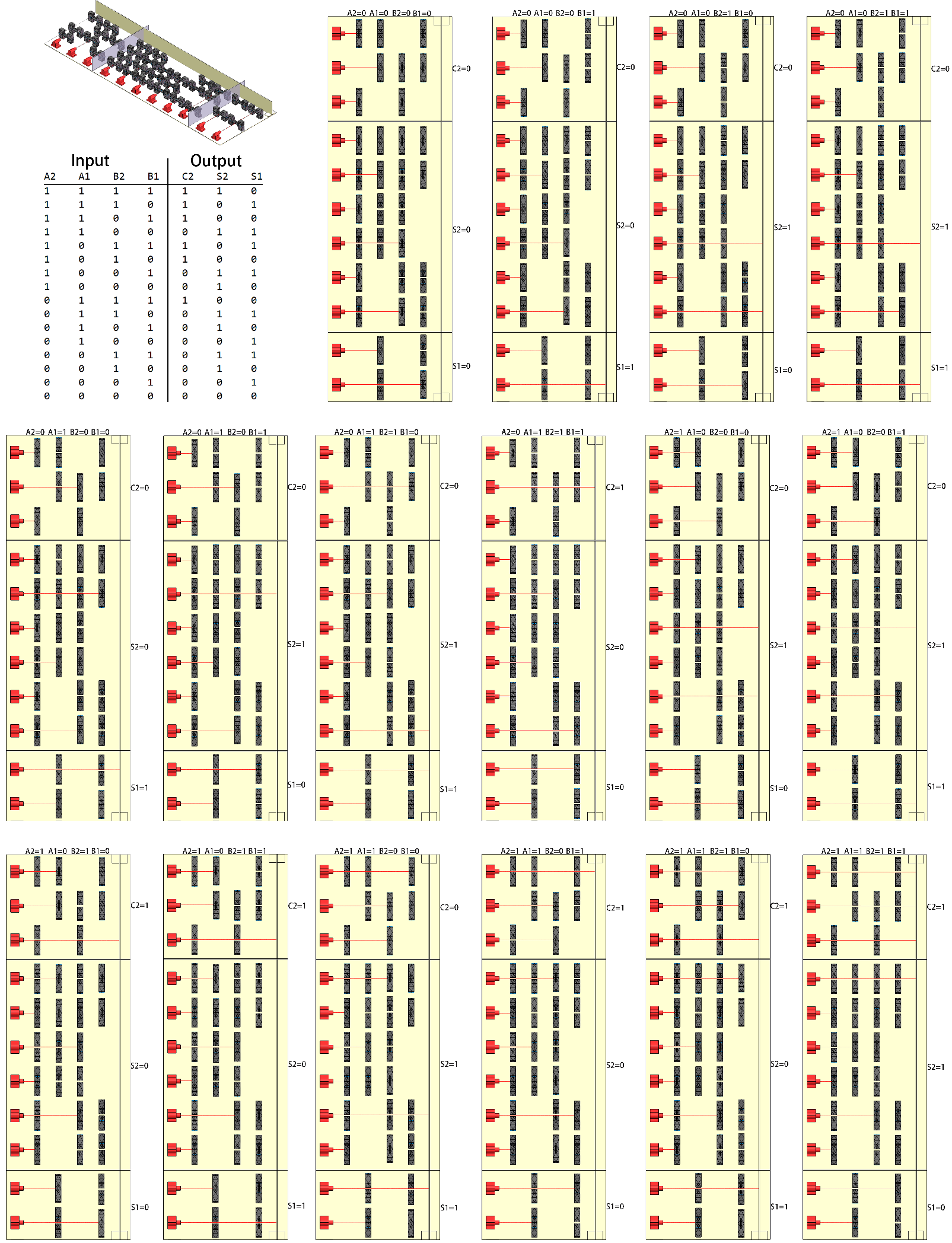


**Fig. S7. Operations of** $\boldsymbol{A}\boldsymbol{2}\left| \boldsymbol{A}\boldsymbol{1}\boldsymbol{\oplus}\mathbf{B}\mathbf{2} \right|\boldsymbol{B}\boldsymbol{1=C}\boldsymbol{2}\left| \boldsymbol{S}\boldsymbol{2} \right|\boldsymbol{S}\boldsymbol{1}$**.** System model, truth table, and simulated operations for 16 cases.


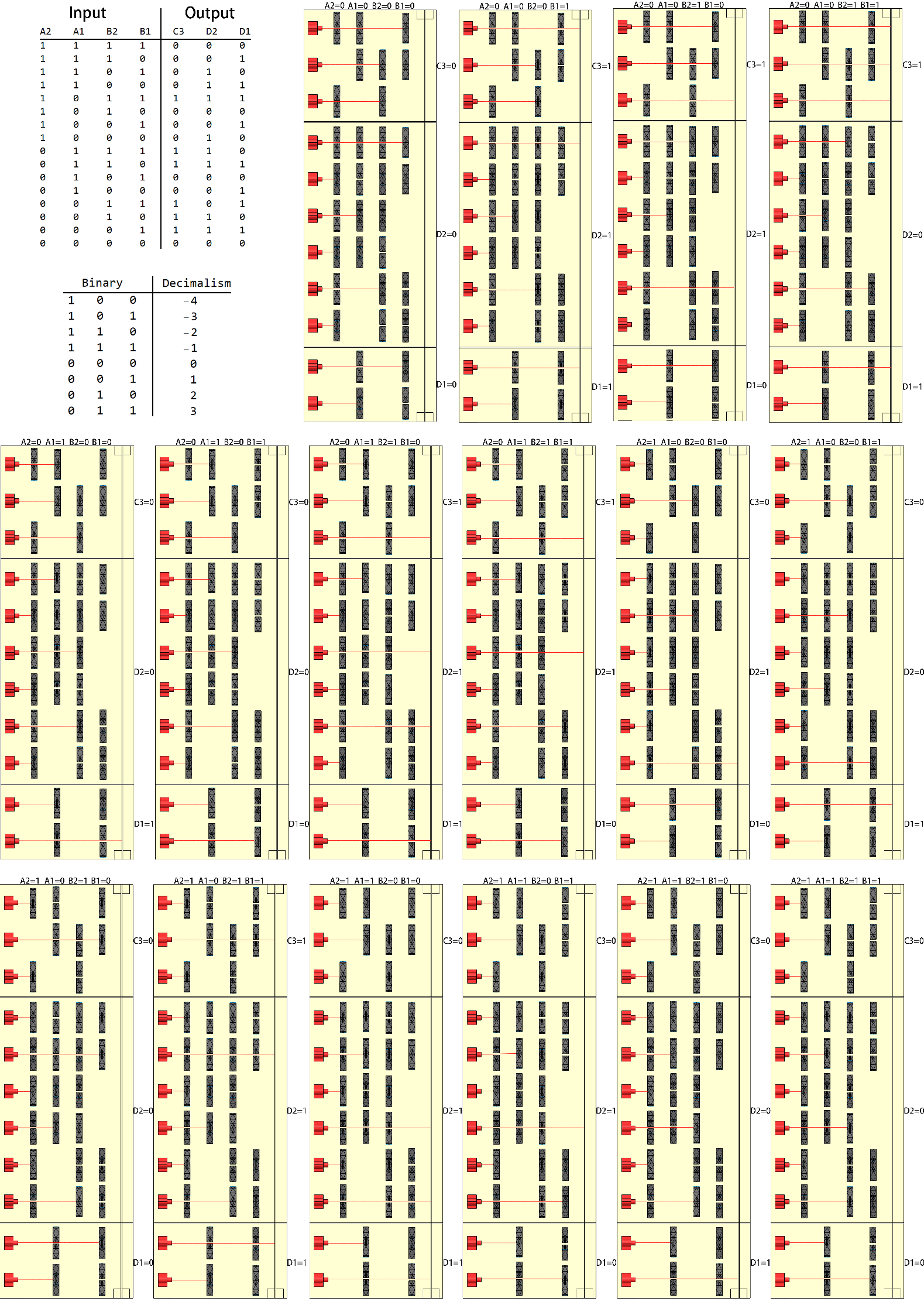


**Fig. S8. Operations of** $\boldsymbol{A}\boldsymbol{2}\left. \boldsymbol{|A}\boldsymbol{1} \mathbf{㊀} \boldsymbol{B}\mathbf{2|} \right.\boldsymbol{B}\boldsymbol{1=C}\boldsymbol{3}\left| \boldsymbol{D}\boldsymbol{2} \right|\boldsymbol{D}\boldsymbol{1}$**.** Truth table, relation between the binary results and decimal numbers, and simulated operations for 16 cases. For $A2\left. |A1\geq B2| \right.B1$, for example, $1\left. |1 ㊀ 0| \right.1=0\left| 1 \right|0$, which is the decimal number 2 from the relation between binary results and decimal numbers. For $A2\left. |A1<B2| \right.B1$, for example, $0\left. |0 ㊀ 1| \right.0=1\left| 1 \right|0$, which is the decimal number -2.

1. **Force vs. z-strain relationships with different friction coefficients**


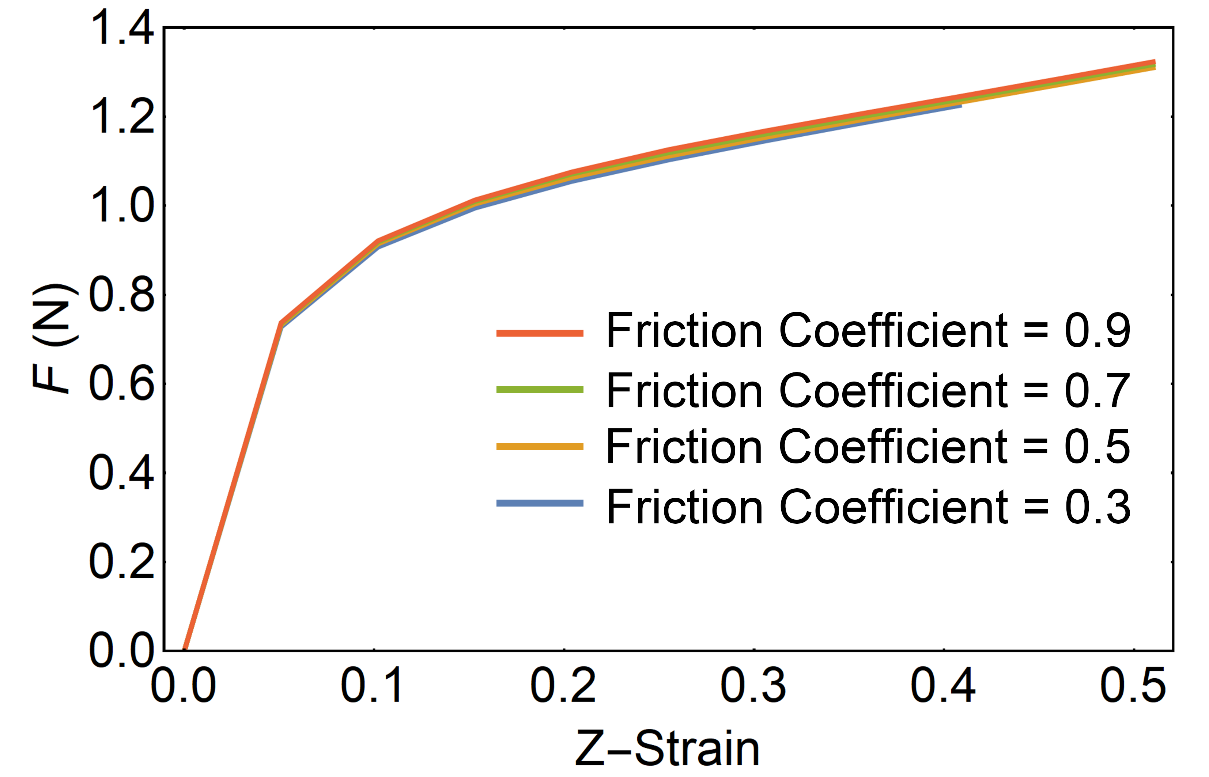


**Fig. S9. Force vs. z-strain with different friction coefficients (simulation).** The friction coefficient influences the final calculation results very slightly. To minimize the error between the calculation and measurement results (force vs. z-strain, see Fig. 1D), friction coefficient of 0.7 is chosen in the main text. Here, friction coefficient of 0.3 cannot make a convergent calculation result near the strain of 0.5.

1. **Supplementary Videos**

Video-1 Soft B-shaped structure compressed to 0.5 strain (compression speed: 15 mm/min)

Video-2 AND gate operation

Video-3 OR gate operation

Video-4 XOR gate operation

Video-5 Half adder operation (A plus B)

Video-6 Half subtractor operation (A minus B)

Video-7 ADC to convert the displacement signal into binary numbers

Video-8 Change of ADC coding scheme to generate disordered numbers
